# Report on the development and application of PET/CT in mainland China

**DOI:** 10.18632/oncotarget.16295

**Published:** 2017-03-16

**Authors:** Yumei Chen, Ruohua Chen, Xiang Zhou, Jianjun Liu, Gang Huang

**Affiliations:** ^1^ Department of Nuclear Medicine, Ren Ji Hospital, School of Medicine, Shanghai Jiao Tong University, Shanghai, China; ^2^ Department of Cancer Metabolism, Institute of Health Sciences, Chinese Academy of Sciences and Shanghai Jiao Tong University School Medicine, Shanghai, China; ^3^ Shanghai University of Medicine & Health Sciences, Shanghai, China

**Keywords:** ^18^F-FDG, general investigation, PET, PET/CT

## Abstract

**Purpose:**

To examine the development and application of systems combining positron emission and x-ray-computed tomography systems (PET/CTs) in mainland China.

**Methods:**

Using a questionnaire, we surveyed Chinese medical institutions on a variety topics relating to their PET/CT systems and its use. The respondents had PET/CTs installed and in clinical use before 31 December 2015. We examined the clinical scenarios to which Chinese PET/CTs were applied by reviewing the related Chinese and international literature from the start of 1995 to the end of 2013; these papers were found by searching the Wanfang and PubMed databases, respectively. The data were then classified and analyzed statistically.

**Results:**

At the end of 2015, there were 240 PET/CTs and 101 medical cyclotrons in mainland China. The total number of PET studies performed in 2015 was 469,364. The main clinical applications of PET were found to be diagnostic fludeoxyglucose (^18^F-FDG) imaging and oncological imaging. A minority of PET/CT studies were performed using ^11^C-choline and other imaging agents. The number of papers relating to clinical use of PET/CT in mainland China increased each year over the period of study, in both the Chinese and international literature. Despite this progress, important problems were also apparent, including unbalanced regional development and the limited quality of the research.

**Conclusions:**

This study provides detailed information for understanding the development PET/CT technology in mainland China, along with its geographical distribution and clinical application. It may thus prove a useful reference for all those involved in planning the future of PET/CT in China.

## INTRODUCTION

The first commercial PET/CT (positron emission tomography/computed tomography) scanner, the *Discovery LS* (GE Healthcare), became available in early 2001 [1]. Clinical use of PET/CTs in China began in September 2002 [2]. Over the last ten years, more than 200 PET/CTs have been installed all over the country. Hundreds of thousands of cases have been acquired, and they are now routinely used for imaging tumors, the cardiovascular system and the nervous system. In addition, thousands of studies using PET/CT have been carried out by medical researchers over this same period. Thus, PET/CT has played an important role in the development and promotion of nuclear medicine in China.

In order to gain a more detailed understanding of the development and application of PET/CT in mainland China, we surveyed Chinese medical institutions and analyzed the related literature. Our purpose was the provision objective data to aid future development of this technology in China.

## RESULTS

### Distribution of PET/CTs in China

**Figure 1:**
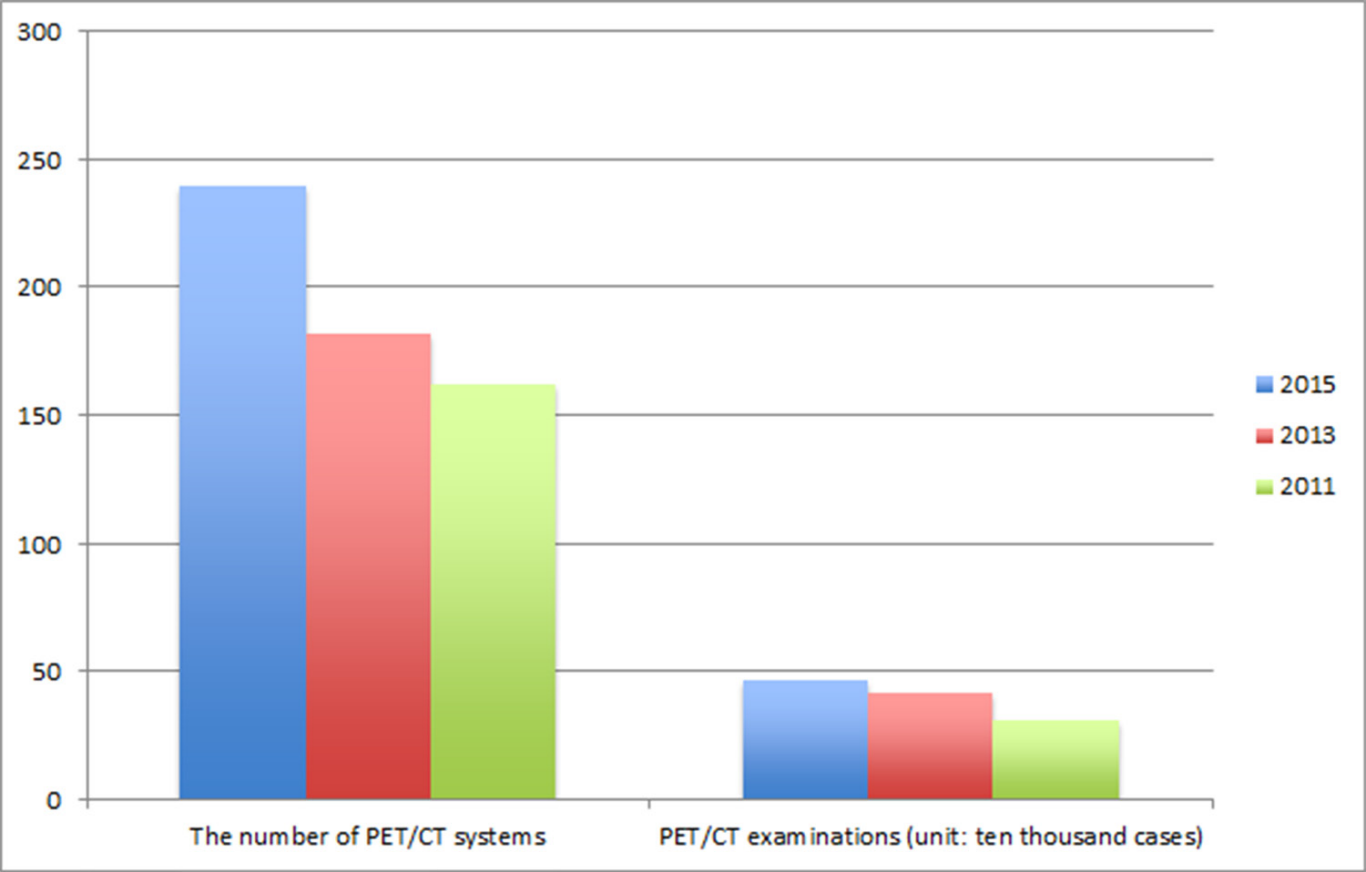
The number of PET/CT systems and PET/CT examinations in mainland China Left: The number of PET/CT systems present in mainland China in 2011, 2013, and 2015. Right: The number of PET/CT examinations carried out in 2011, 2013, and 2015.

Our present investigation indicates that there were 240 PET/CTs (including PET and PET/CT) in mainland China at the end of 2015 (Figure 1), and that they were distributed across 229 medical institutions. Large medical institutions hosted 95.2% of these systems and 65.94% of all PET/CTs were under the jurisdiction of the institution's nuclear medicine department; with 14.85% under radiology, 13.54% under an independent PET/CT center, and 5.67% under another jurisdiction. For medical cyclotrons, the number in mainland China was found to be 101.

The previous national investigation, carried out in 2014, showed that there were 182 PET/CTs in mainland China at the end of 2013, and that these were distributed across 178 medical institutions and throughout 90.6% of the provinces. Large medical institutions hosted 94.6% of the systems, with 64.89% under the jurisdiction of nuclear medicine, 10.11% under radiology, 19.15% under an independent PET/CT center and 5.85% under another jurisdiction. The hospital managed 88.83% of PET/CTs; 10.11% were jointly managed by the hospital and investors and 1.06% were managed by investors.

### Cost and reimbursement

The majority of PET/CTs were jointly funded by the state and hospitals, and the rest were funded by companies. The total cost of a PET/CT examination ranged from 7,000 to 10,000 RMB. Fees for technical aspects and those for the physician's interpretation were not separated.

### Training of physicians and technicians

PET/CT physicians and technicians were trained by a medical college and the affiliated hospital. In addition to a clinically relevant qualification, PET/CT physicians had to obtain a nuclear medicine physician certificate and a CT mount guard card. Technicians required a nuclear medicine technologist certificate. We found that there are now many PET/CT courses available for training of relevant personnel.

### Applications of PET/CT

Our study indicated that the total number of PET examinations performed in mainland China in 2015 was 469,364, an increase of 4.4% compared with 2013 (Figure 1). Among these examinations, cancer accounted for 86.9%, while cardiovascular diseases accounted for 0.8%, neurological diseases for 2.7%, physical examinations for 6.5%, bone imaging for 0.2%, and other purposes accounted for 2.9%.

Our 2014 investigation showed that the total number of PET examinations performed in mainland China in 2013 was 418, 744. Among these, cancer accounted for 81.12%, while cardiovascular and neurological diseases accounted for 0.97% and 2.19%, respectively. The remainder comprised of studies employing PET/CT for physical examinations (13.35%) or others purposes (1.94%).

### Quantity of papers in the science citation index (SCI)

**Figure 2:**
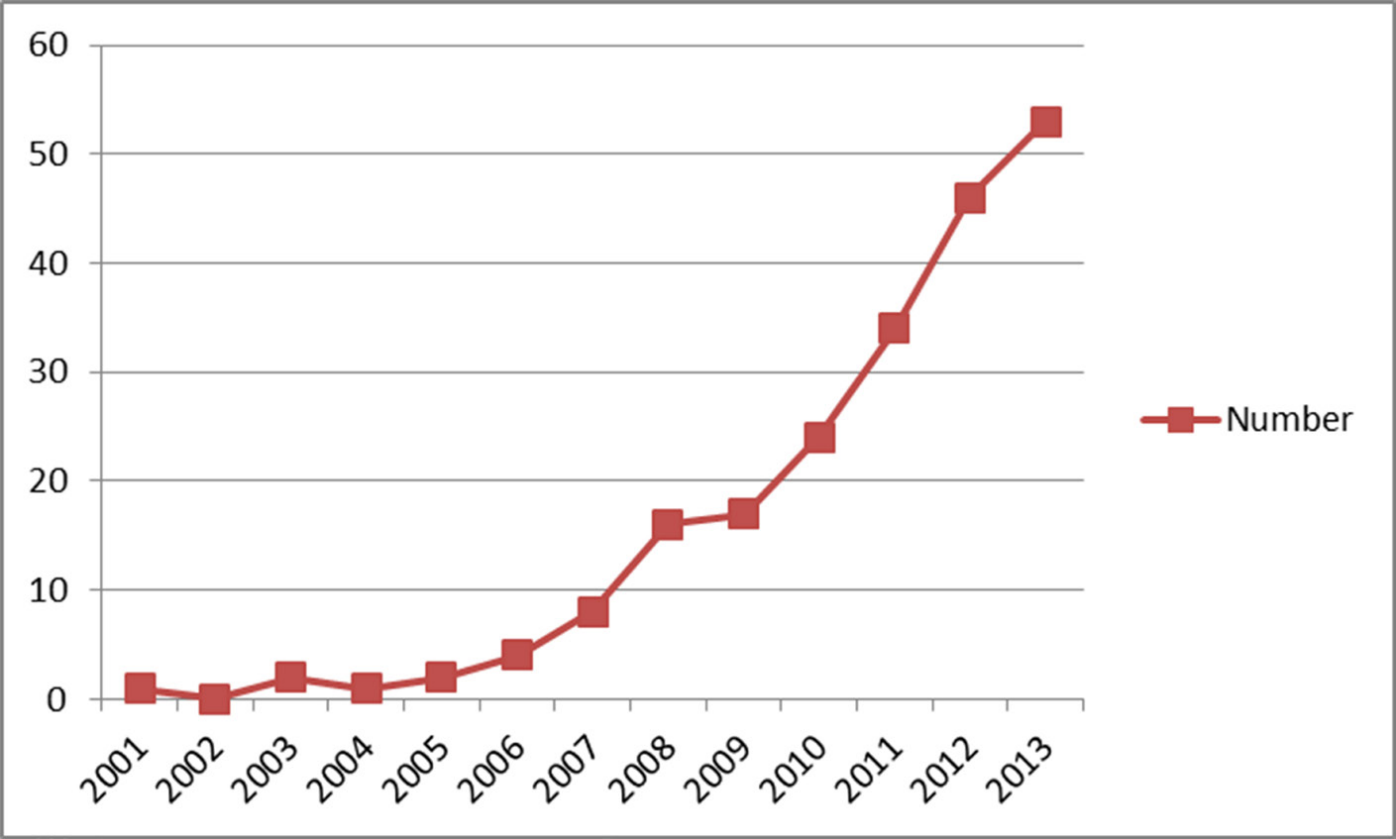
The number of SCI (the Science Citation Index) papers involving clinical use of PET/CT in mainland China from 2001 to 2013

For the English-language papers included in the study, we examined their occurrence in the SCI. The earliest is one published in the *CHINESE MEDICAL JOURNAL* in 2001 [3], and from this time on, the number of indexed papers (SCI papers) increases year on year, reaching 208 by the end of 2013 (Figure 2).

### Quality of SCI papers

**Figure 3:**
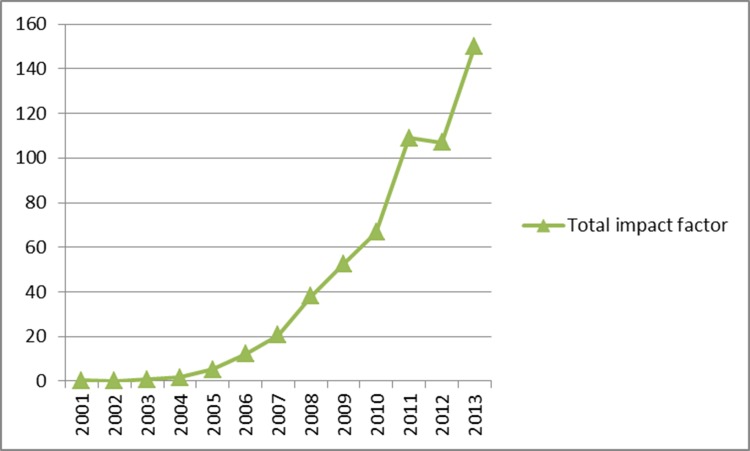
Total impact factor of SCI papers involving clinical use of PET/CT in mainland China from 2001 to 2013

**Figure 4:**
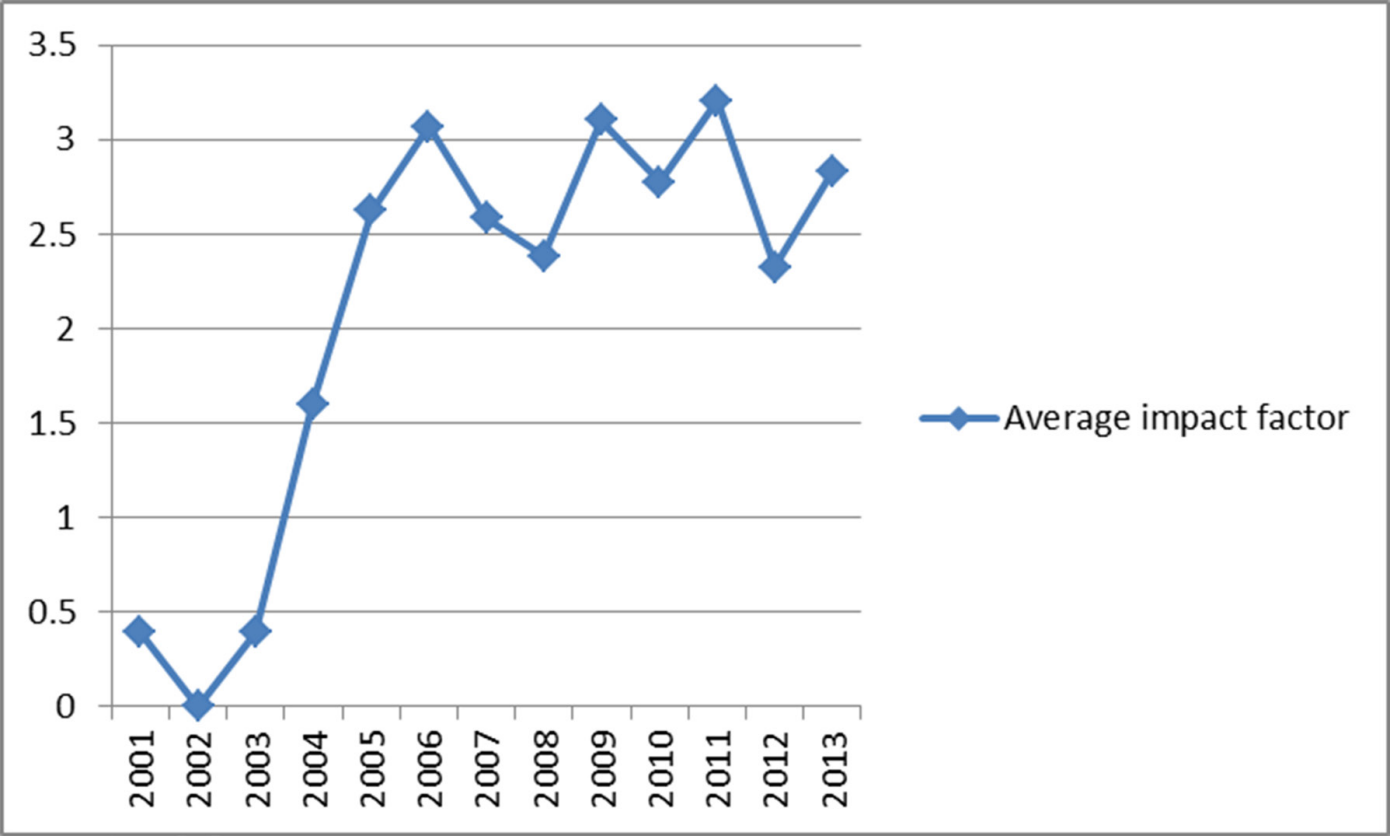
Average impact factor of SCI papers involving clinical use of PET/CT in mainland China from 2001 to 2013

The total impact factor of the SCI papers was 544.277, and the average was 2.713. The total impact factor increased each year (except 2012) between 2001 and 2013 (Figure 3). The average impact factor increased approximately 6-fold between 2003 and 2005, but did not change substantially thereafter (Figure 4). The ten journals with greatest numbers of papers within the remit of our study were as follows: 20 in Clinical Nuclear Medicine, 16 in the Journal of Nuclear Medicine, 16 in Nuclear Medicine Communications, 15 in the Chinese Medical Journal, 8 in the International Journal of Radiation Oncology Biology Physics, 7 in the European Journal of Radiology, 7 in Annals of Nuclear Medicine, 6 in PLoS One, 5 in the World Journal of Gastroenterology, and 4 in each of the following, Lung Cancer, the European Journal of Nuclear Medicine and Molecular Imaging, Academic Radiology and the Asian Pacific Journal of Cancer Prevention.

### Regional distribution of home institutions for SCI papers

**Figure 5:**
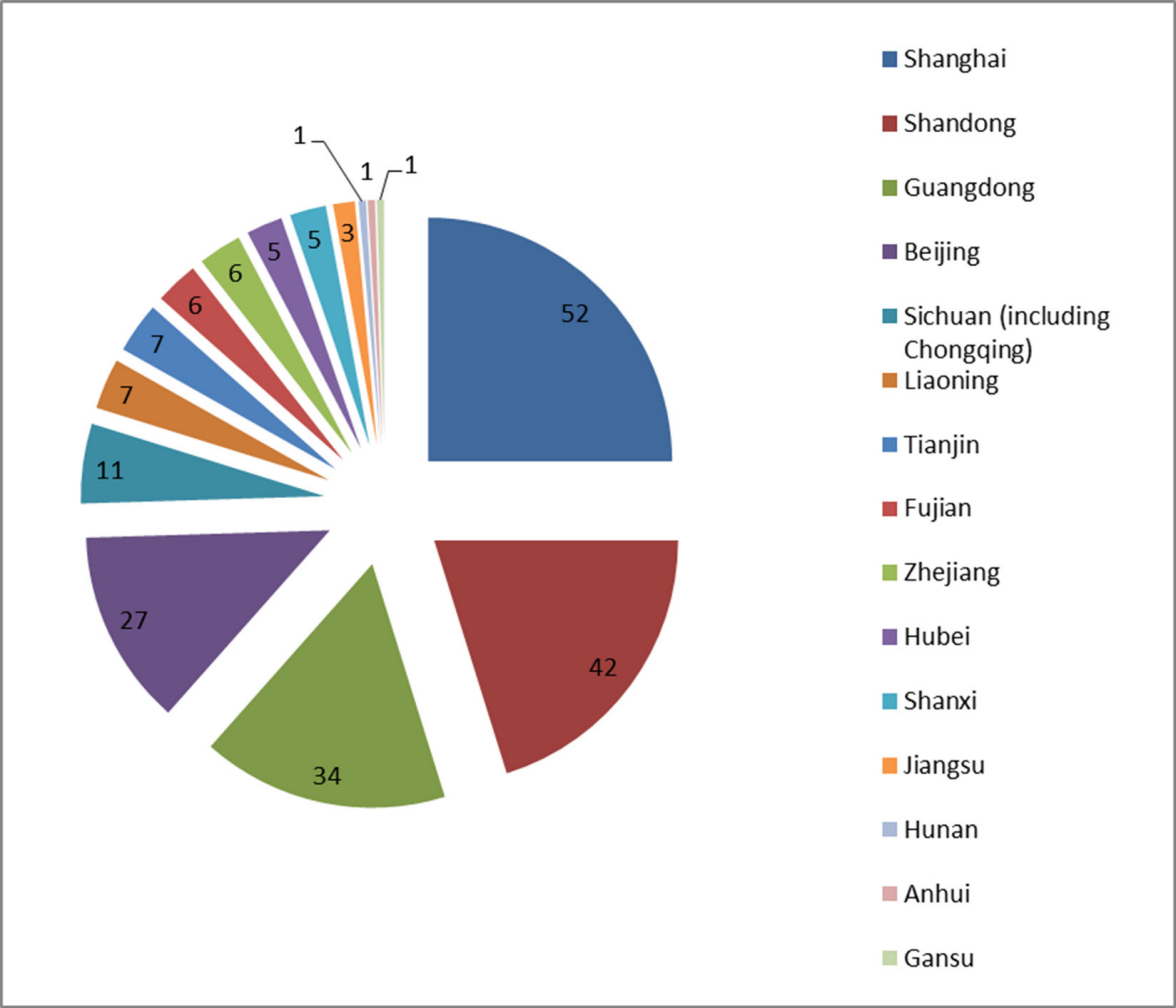
Regional distribution of author institutions for SCI papers involving clinical use of PET/CT in mainland China at the end of 2013

According to the number of papers and the affiliation of the first author, the top five geographical regions were Shanghai, Shandong, Beijing, Guangdong and Sichuan (including Chongqing; Figure 5). Based on number of papers, the top ten institutions were Shandong Provincial Cancer Hospital (29), Shanghai Cancer Hospital (17), Huashan Hospital (14), Beijing General Hospital of People's Liberation Army (13), Zhongshan University Cancer Research Center (9), State Key Laboratory of Oncology in South China (9), Nanfang Hospital (8), Shangdong Province-Owned Hospital (7), the First Affiliated Hospital of Zhongshan University (7), Ruijin Hospital (6) and Tianjin Cancer Hospital (6).

### Content of SCI Papers

**Figure 6:**
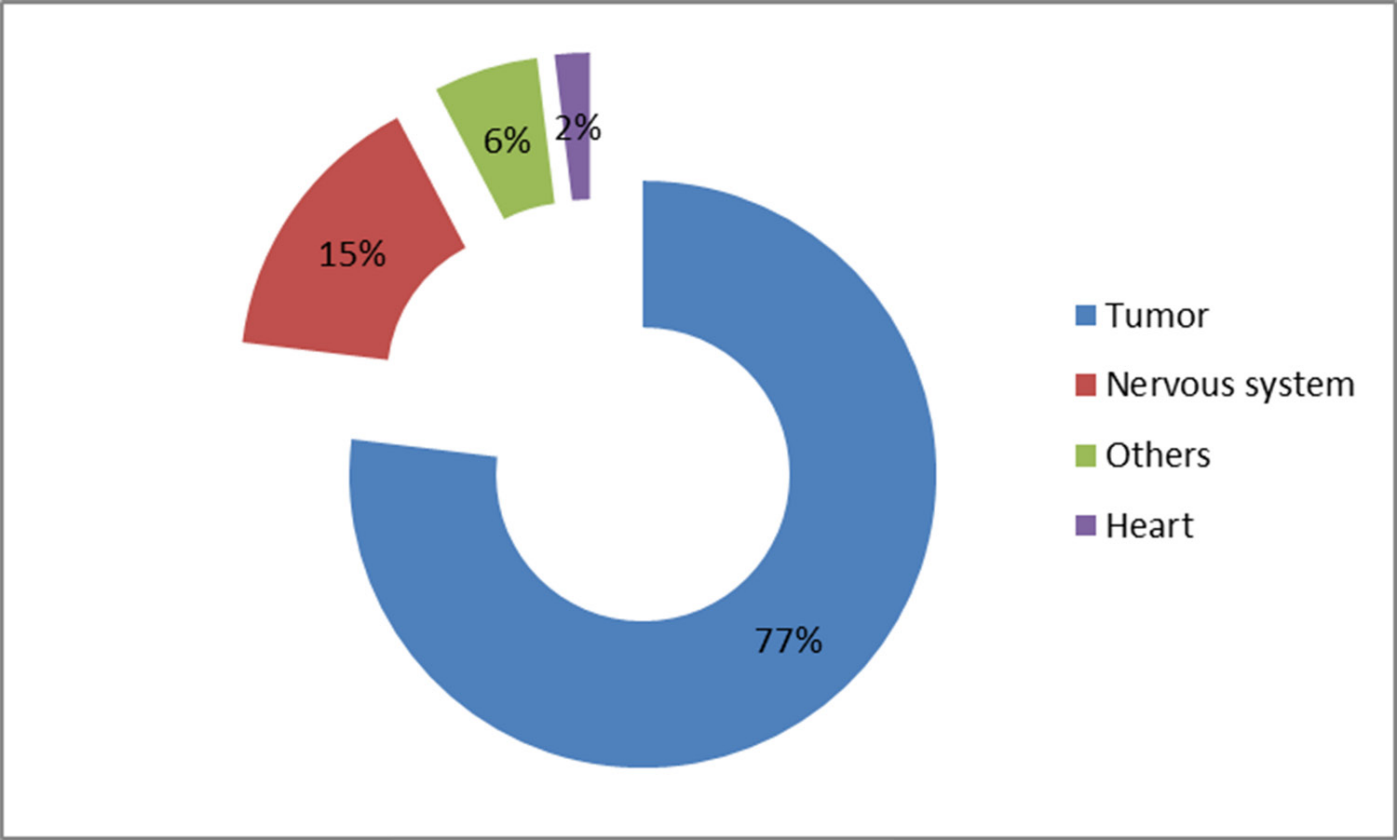
Distribution of research topics for SCI papers involving clinical use of PET/CT in mainland China at the end of 2013

Of the 208 papers, 160 (77%) described cancer studies, 32 (15%) described neurological studies, 4 (2%) described cardiac imaging and 12 (6%) described other research topics (Figure 6). The following clinical applications were described among the cancer studies: tumor diagnosis and staging (74); prognosis evaluation (21); prediction of recurrence, therapeutic evaluation and re-staging (19); guidance of clinical decision making and treatment planning (18); tumor behavior and metabolism (14); and finally, tumor metabolic imaging and assessment methodologies (14). The five most frequently studied cancers were lung (40), esophageal (18) and gastrointestinal (16) tumors, nasopharyngeal carcinoma (10), and lymphoma (9).

Forty of the 208 papers involved to clinical research based on non-fludeoxyglucose (non-FDG) imaging agents, with the top five agents being ^11^C-choline (11), ^18^F-fluorothymidine (^18^F-FLT; 10), ^13^N-ammonia (4), ^11^C-2β-carbomethoxy-3β-(4-fluorophenyl)tropane (^11^C-CFT; 3) and ^18^F-fluoroerythronitroimidazole (^18^F-FETNIM3; 3). The imaging agents and clinical applications are given in Table 1.

**Table 1:** Imaging agents and clinical applications

Imaging agents	Imaging mechanism	Clinical applicatio	Number of SCI papers
11C-cholin	Tumor proliferation	Lung cancer, central nervous system tumors, nasopharyngeal carcinoma, and thyroid carcinoma	11
18F-FLT	Tumor proliferation	Lung cancer, esophageal cancer, and gastroenteric tumor	10
13N-ammonia	Diffusion	Glioma of central nervous system and lymphoma	4
18F-FETNIM	Hypoxia	Lung cancer and esophageal cancer	3
11C-CFT	Dopamine transporter	Parkinson's disease	3
11C-PD153035	EGFR	Lung cancer	2
18F-HX4	Hypoxia	Head and neck neoplasm	1
18F-FP-CIT	Dopamine transporter	Alzheimer disease	1
11C-methionine	Amino acid tracer	Glioma	1
11C-NMSP	Monoamine receptor	Brain function	1
18F-FES	Estrogen receptor	Breast cancer	1
18F-alfatide	Expression of αvβ3	Lung cancer	1
18F-FMISO	Hypoxia	Breast cancer	1

In 92 of the SCI papers, the first author was from a nuclear medicine department, PET center or a radiology department, and the remaining 116 first authors were from clinical departments.

### Quantity of Papers in Chinese Journals

**Figure 7:**
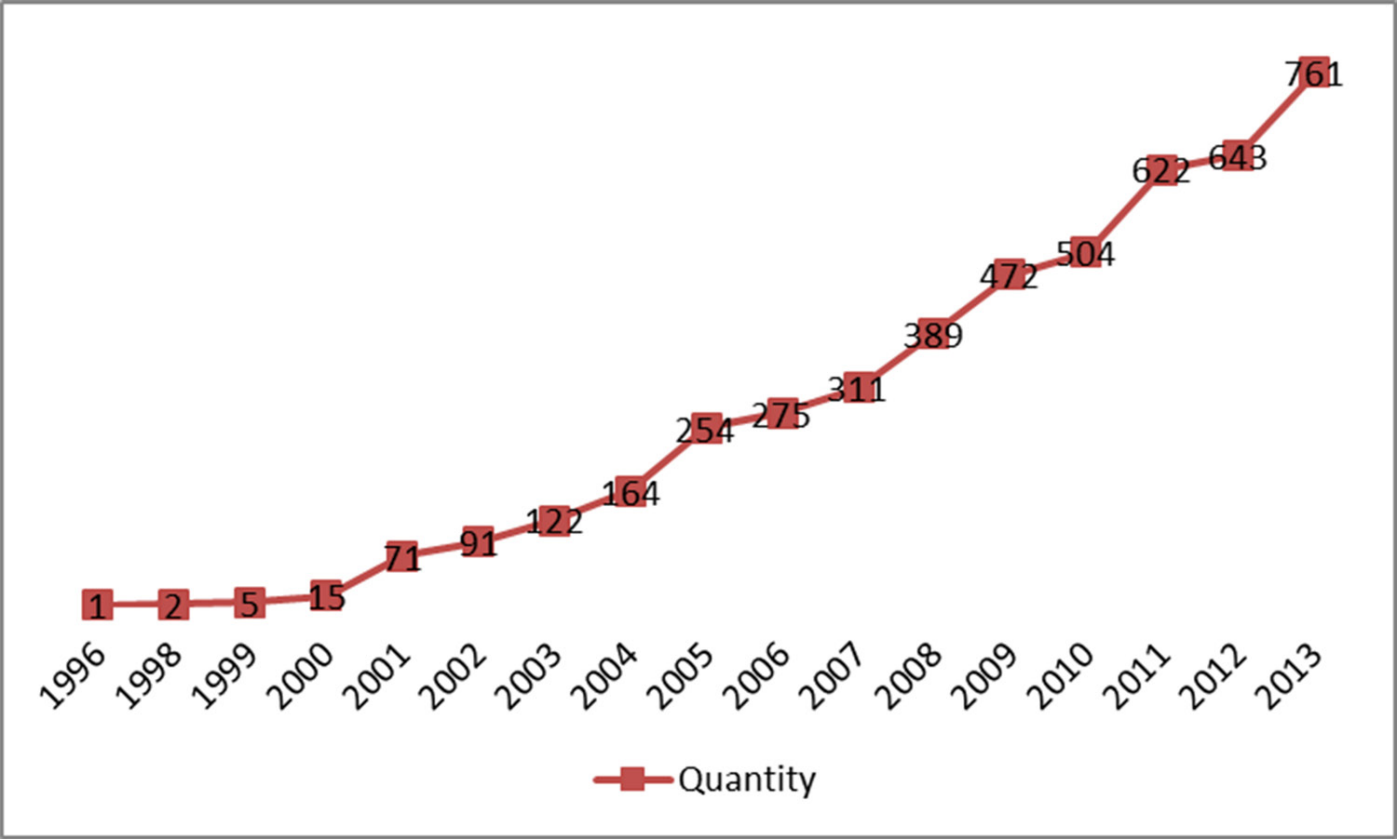
The number of papers from Chinese publishers that involve clinical use of PET/CT in mainland China (as of 31 December 2013)

There were 4,702 papers on PET published by Chinese journals between 2001 and 2013, with substantial increases occurring each year over this period (Figure 7).

**Figure 8:**
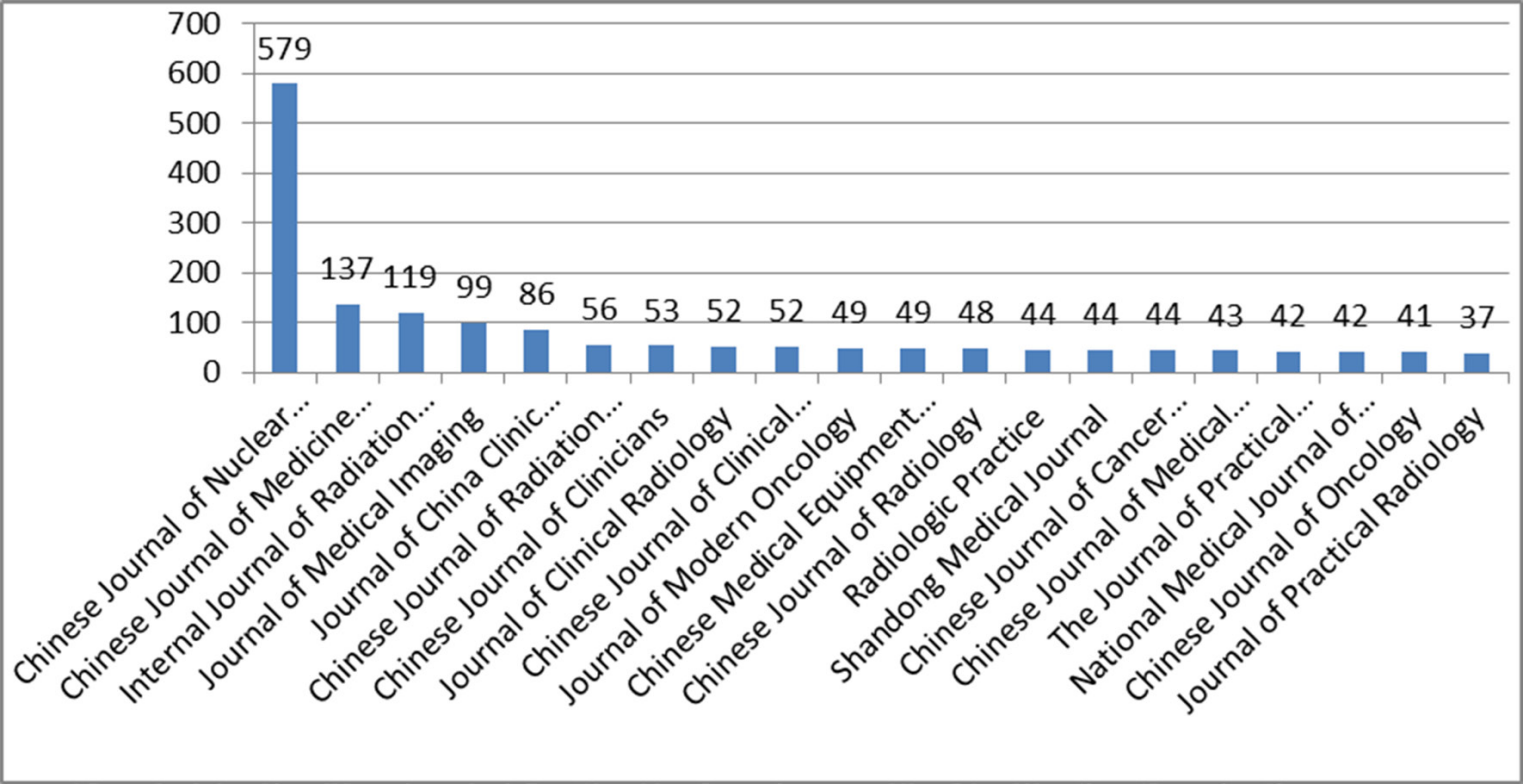
The top twenty Chinese journals, according to the quantity of PET-based clinical papers published

By far the most frequent publisher among this group, was the Chinese Journal of Nuclear Medicine and Molecular Imaging, with 579 papers and 12.3% of the total. The top twenty Chinese journals, according to the quantity of PET papers published, are shown in Figure 8.

### Content of papers in the Chinese Journal of Nuclear Medicine and Molecular Imaging

**Figure 9:**
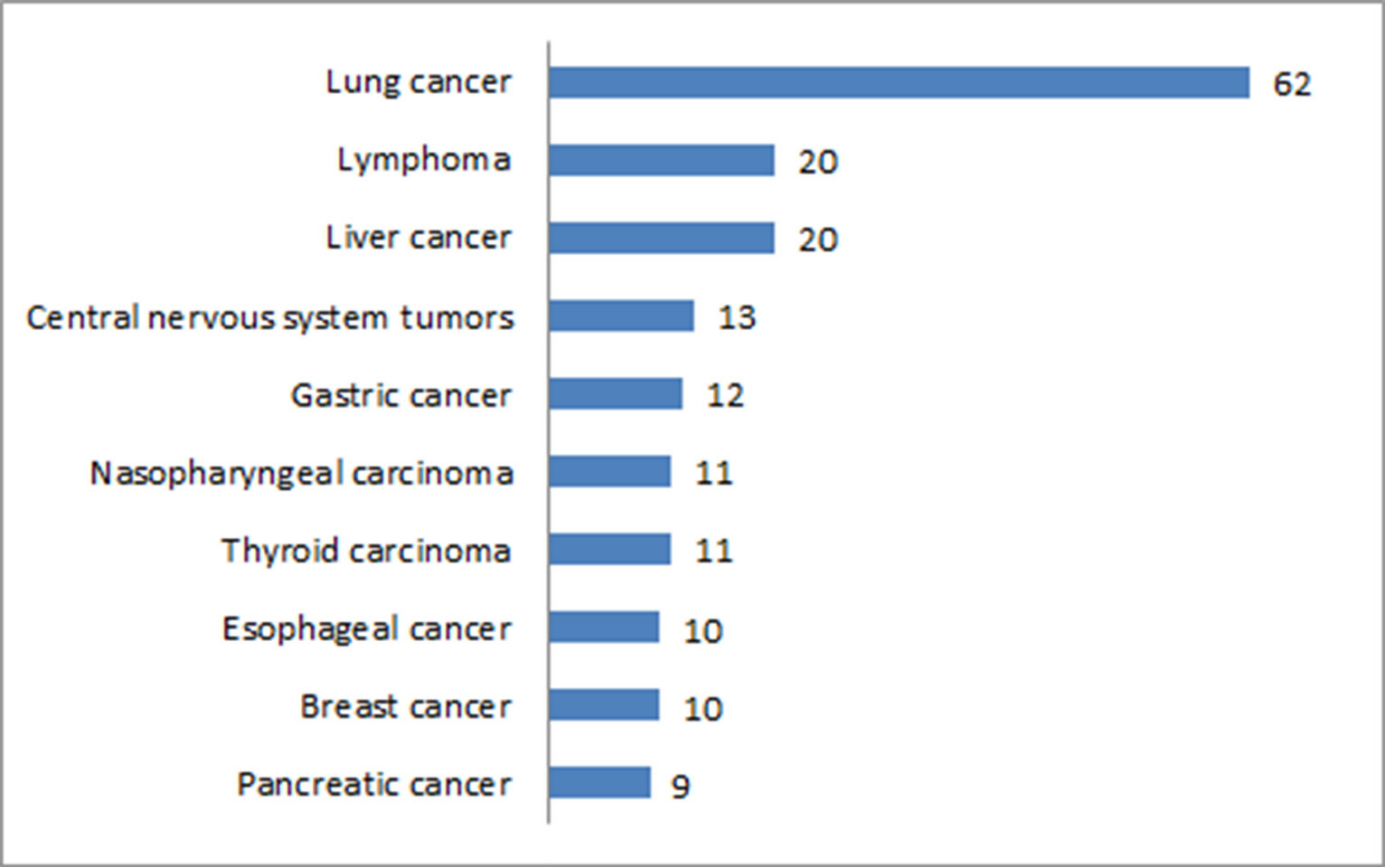
Top ten cancers under study in PET-based clinical papers from the Chinese Journal of Nuclear Medicine and Molecular Imaging

We examined the contents of papers in the Chinese Journal of Nuclear Medicine and Molecular Imaging, the most authoritative Chinese journal on nuclear medicine and the one with the most PET-based clinical papers (Table 2). Papers on cancer accounted for 49.6% (287/579). Within this group, the top ten cancers under study were lung cancer, lymphoma, liver cancer, central-nervous-system tumors, gastric cancer, nasopharyngeal carcinoma, thyroid carcinoma, esophageal cancer, breast cancer and pancreatic cancer (Figure 9). There were 404 papers that used FDG as an imaging agent, and 105 papers that used other imaging agents.

**Table 2:** Research topics covered by PET-based papers in the Chinese Journal of Nuclear Medicine and Molecular Imaging

Research contents	Quantity
Tumor	287
Diagnosis and differential diagnosis	125
Therapeutic evaluation, prognosis evaluation	42
Staging, re-staging	16
Adjuvant radiotherapy	12
Methodological study	38
Other aspects of tumor	54
Nervous system	77
Heart	31
Others	184

## DISCUSSION

Today, PET/CT is the most mature and widely used clinical molecular imaging technology. Development and expansion of China's PET/CT facilities and associated clinical practices has enhanced its ability to provide high-quality medical care and to compete internationally on this front. The technique provides a new means for diagnosis and treatment of cancer, and promotes the interplay of multiple disciplines and the development of medical science.

Although there has been a rapid rise in the number of Chinese PET/CT centers (increasing nearly every year since 2001), whether their full clinical potential is being realized has remained unclear. For this purpose, we surveyed and analyzed the development and application of PET/CT in mainland China by means of a questionnaire and a literature review. We sought to obtain an objective and accurate understanding of the development of PET/CT in China, to provide a sound basis on which to optimize its use across the country.

Our results showed that the demand for and quantity of PET/CTs is increasing in China. When compared to those for 2013, the 2015 values indicate respective increases of 31.9% and 4.4% for the number of PET/CT systems in operation and the number of PET/CT examinations carried out in the year. The number of research papers on clinical use of PET/CT increased steadily from 2001 to 2013, but the total number and quality of SCI papers from China was substantially lower than corresponding values for papers from the United States and other developed countries. As would be expected, the total impact factor of the Chinese SCI papers increased with the total number of such papers, but the average impact factor appeared static; suggesting that the impact and/or quality of PET/CT-based clinical research remains relatively low in China.

Most of these Chinese SCI studies exhibited problems with experimental design, accuracy of data acquisition and/or presentation, and how comprehensively they addressed the issue under investigation. However, there were some examples of high-quality, high-impact Chinese papers in the SCI, such as one authored by researchers from the Zhongshan University Cancer Center and titled, “Prospective Study on the Staging of Nasopharyngeal Carcinoma, Clinical Decision and Economic Benefit through FDG PET/CT and Epstein-Barr Virus DNA” [4]. This paper is a valuable reference for clinicians in the field, and it was deservedly published in the authoritative and high-impact Journal of Clinical Oncology.

Another factor contributing to the relatively low impact of Chinese research in this field is the fact that multi-center studies are still rare in China; only 3 of the 208 papers were based on multi-center studies. They were “Research on FDG PET in Staging of Lung Cancer-lymph Node” by authors at the Shandong Provincial Tumor Hospital, “Research on Cerebral Metabolism in Alzheimer's Disease” from the Chinese Academy of Sciences, and “Research on Differential Diagnosis of Pulmonary Nodule by Dual isotope Simultaneous Acquisition FDG and FLT” from the Beijing General Hospital of People's Liberation Army [5–7]. These papers were all published in high-quality journals, thus showing that multi-center studies can provide a means to increase the quality of research.

Regional imbalances in how PET/CTs are distributed and used is a problem for China. The systems are mainly found in developed regions, such as Beijing, Shanghai, Guangdong and Shandong, and the situation is similar for PET/CT-related clinical research, which is concentrated in Shanghai, Shandong, Guangdong and Beijing. In some areas, the institutions have not fully exploited the potential of their equipment, while other factors include differences in regional economic development, and regional differences in the cost of PET/CT examinations versus patient income. More balanced future development of PET/CT in mainland China will require a number of systemic changes including increased cooperation between institutions, standardized professional training, strictly implemented quality control and best practices, active promotion of PET/CT coverage by medical insurance, and encouragement of physicians and technicians to maximize the clinical and research benefits of this expensive medical resource.

Despite the current imbalances, PET/CT facilities are gradually spreading to the smaller cities. However, this positive trend has spawned another problem, a shortage of physicians, radiopharmacists, radiochemists and medial physicists in these regions. To address this, medical schools are expanding enrollment into the corresponding professional training programs. Furthermore, the physicians currently specializing in imaging are being encouraged to train in PET/CT.

From the results of the survey and literature review, it was found that PET/CT was mainly used for metabolic imaging of tumors. The value of PET/CT in diagnosis, staging, therapeutic evaluation and prognosis has been fully demonstrated in the literature [8,9]. We found that the first authors of 55.8% of the SCI papers were from clinical departments, indicating the demand for PET/CT in clinical practice. Clinicians now have a deeper understanding of the value of PET/CT. The proportion of SCI papers concerned with clinical application of PET/CT to the nervous system and cardiac metabolism was low, especially for the latter. Cardiac metabolic imaging was only conducted in a few specialist hospitals, and the vast majority of hospitals with PET/CTs hardly ever carried out such procedures. Cancer imaging studies mainly focused on high-incidence diseases, such as lung cancer, esophageal cancer, gastrointestinal cancer, nasopharyngeal carcinoma and lymphoma [10–14]. A large majority of the SCI papers, 80.8%, used FDG as the imaging agent, but some used other agents such as ^1^C-choline and ^18^F-fluorothymidine [15–17]. Therefore, there is much room for future development in non-cancer-related imaging and in the clinical exploitation of new imaging agents; all of which would be aided by improved interdisciplinary communication and cooperation.

In conclusion, our results showed that there were 240 PET/CTs and 101 medical cyclotrons in mainland China at the end of 2015. The total number of PET/CT examinations carried out in China that year was 469,364. Over the period from 2001 to 2013, clinical PET was mainly used for diagnostic ^18^F-FDG imaging and for oncological imaging; other imaging agents such as ^11^C-choline were also used, but only in around 20% of cases. The quantity of papers involving clinical use of Chinese PET/CTs was found to increase year after year, for both national and international (SCI) publications. However, our study also indicated some important problems for PET/CT in China today, particularly the unbalanced regional development of PET/CT facilities and the low quality of PET/CT-based research.

## MATERIALS AND METHODS

### Survey

The Molecular Imaging and Nuclear Medicine Branch of the Chinese Medical Association conducted this national investigation during January and February of 2016. A questionnaire was sent out to all Chinese medical institutions and all respondents represented institutions with PET/CTs (including PET and PET/CT) or medical cyclotrons in clinical use before 31 December 2015. The section of the questionnaire concerning the use of PET/CTs and medical cyclotrons, as well as related practices, was divided into six subsections that covered 46 questions. The section investigating general aspects of the PET/CT facility was divided into 9 subsections comprising a total of 65 questions, which concerned the basic information of the medical institution and department, membership and setting, installation and use of main equipment, radionuclide therapy projects and their quantity, *in vitro* and functional testing, staff composition, teaching, and any suggestions the respondent might have for the medical association. The investigation form can be downloaded from www.chinanm.org.cn (in Chinese) after registration. The statistical data covered 31 provinces (municipalities and autonomous regions) in mainland China. The obtained data was compared with that of the 2014 national investigation, in which case, all respondents represented institutions with PET/CTs or medical cyclotrons in clinical use prior to 31 December 2013.

### Literature review

PubMed was used to retrieve English-language papers with the keyword “positron emission tomography” and then, after carefully reading the author affiliations, title, abstract and full text, we selected those that concerned clinical applications of PET or PET/CT conducted within mainland China. Case reports, review articles and meta-analyzes were not included. If the paper's first author was Chinese, but the data in the paper was from a study conducted outside of mainland China, it was also excluded. The Wanfang database was used for retrieving Chinese-language papers. We searched for the keyword “PET” and recorded the title, journal, publication year, author affiliations and research content of the resulting papers. Only those published prior to 31 December 2013 were considered.

## Abbreviations

PET/CT: positron emission tomography/computed tomography
SCI: the Science Citation Index
